# Exploring the Impact of the Gut Microbiome on Obesity and Weight Loss: A Review Article

**DOI:** 10.7759/cureus.40948

**Published:** 2023-06-25

**Authors:** Jawad Noor, Ahtshamullah Chaudhry, Saima Batool, Riwad Noor, Ghulam Fatima

**Affiliations:** 1 Internal Medicine, St. Dominic Hospital, Jackson, USA; 2 Pathology, Nishtar Medical University, Multan, PAK; 3 Medicine/Public Health, Nishtar Hospital, Multan, PAK; 4 Internal Medicine, Abbasi Shaheed Hospital, Karachi, PAK

**Keywords:** synbiotics, probiotics, prebiotics, weight loss, gut microbiome, obesity

## Abstract

The global obesity pandemic has prompted efforts to search for novel intervention options, including maximizing the health benefits of certain gut microbes and their metabolic byproducts. Our increased understanding of gut microbiota can potentially lead to revolutionary advancements in weight management and general well-being. We studied the association between gut microbiota and obesity, as well as the possible benefits of probiotics, prebiotics, and synbiotics in the prevention and management of obesity in this review. We observed a relationship between the metabolism of nutrients, energy consumption, and gut flora. Numerous mechanisms, including the synthesis of short-chain fatty acids, hormone stimulation, and persistent low-grade inflammation, have been postulated to explain the role of gut bacteria in the etiology of obesity. It has been discovered that the diversity and composition of the intestinal microbiome vary in response to various forms of obesity therapy, which raises concerns about the potential impact of these changes on weight loss. According to research, probiotics, prebiotics, and synbiotics may alter the release of hormones, neurotransmitters, and inflammatory factors, thereby diminishing the stimuli of food consumption that lead to weight gain. More clinical research is required to determine the optimal probiotic, prebiotic, and synbiotic supplementation dosages, formulations, and regimens for long-term weight management and to determine how different gastrointestinal microbiome bacterial species may influence weight gain.

## Introduction and background

Obesity is a complex metabolic disorder with an unknown exact etiology that may be caused by a combination of genetic and environmental factors. The World Health Organization (WHO) defines obesity as having a body mass index (BMI) greater than 30, although this definition varies from nation to nation [1]. By 2030, it is estimated that 1.12 billion people worldwide will be obese [2]. According to CDC, the prevalence of obesity in the United States increased from 30.5% in 1999-2000 to 41.9% in March 2020, with severe obesity rates rising from 4.7% to 9.2% during the same period. Recent studies have demonstrated that an imbalance in the intestinal flora may contribute to obesity. The gut microbiota, which numbers up to 100 trillion, feeds on dead cells, intestinal mucus, and residual food not digested by the human body [1].

The term "microbiome" refers to the ecological community of microorganisms that reside in the human intestine and have evolved alongside the host body over thousands of years, forming intricate and beneficial connections [3]. There are approximately 10^13^ microorganisms in the digestive system, with the majority being anaerobic bacteria. It is believed that the combined genomes of the 500-1000 distinct species of these microorganisms contain 100 times as many genes as the human genome [4]. Because of the work of the Human Microbiome Project, the gastrointestinal microbiota, which includes Firmicutes, *Bacteroides*, *Proteus*, Actinomycetes, *Fusobacteria*, and Verrucomicrobia, have been extensively studied [1]. These are the predominant microorganisms found in the intestines of a healthy individual. They also produce short-chain fatty acids (SCFA), enrich lipopolysaccharide (LPS), produce vitamins (A, B, C, K), and synthesize essential amino acids [5]. A diverse and robust composition that is capable of reestablishing equilibrium after disturbances is indicative of a healthy intestinal microbiota. Various factors such as diet, age, and antibiotics can cause changes in the structure and metabolism of the gut microbiota, which in turn may impact energy metabolism and the absorption of nutrients [4]. The active intestinal microbiota produces numerous physiologically active chemicals, such as SCFA, vitamins, and amino acids, while also having the potential to produce harmful substances such as neurotoxins, carcinogens, and immunotoxins [6]. The gut microbiota increase food intake by modulating brain function through various mechanisms involving endocrine, immune, and neural pathways [3].

This review explores the relationship between obesity and gut microbiota in order to better comprehend how the gut microbiome promotes weight gain. This review will also examine how the consumption of probiotics, prebiotics, and synbiotics affects a person's weight.

## Review

Relationship between obesity and the gut microbiome

Obesity has become a global pandemic as a result of sedentary lifestyles and overeating [7]. The gastrointestinal microbiota is essential for digestion, vitamin synthesis, and metabolic function. Some studies suggest that the intestinal microbiota influences the composition of adipose tissues, promotes low-grade inflammation, and enhances the extraction of energy from food, all of which may be contributing to obesity.

Energy Harvest from Diet

The gut microbiome not only influences bile acid signaling, resulting in variations in bile acid profiles, but it also plays a role in lipid and carbohydrate metabolism. Approximately 5-10% of bile acids are transformed by anaerobic intestinal bacteria such as *Bacteroides*, *Eubacterium*, and *Clostridium* [8]. This demonstrates how the microbiota in the intestine affects the digestion and metabolism of dietary lipids. In addition, germ-free mice that received gut microbiomes from obese individuals exhibited higher expression of genetic markers associated with essential amino acid pathways, indicating a link between the gut microbiome and amino acid metabolism. Dietary factors can induce changes in the gastrointestinal microbiota, and while specific dietary interventions for enhancing microbiota diversity and metabolic state may vary between individuals, it is essential to consume a well-balanced diet with adequate daily protein from a variety of food sources [9]. This is significant because nitrogen is required for microbial growth, carbohydrate assimilation, and the synthesis of SCFA.

Polysaccharides and proteins that are not metabolized in the small intestine are fermented into acetate, propionate, and butyrate in the colon by intestinal microbiota. According to estimates, SCFA may supply up to 10% of daily energy requirements and up to 70% of the energy required for colonic epithelial cells to breathe [10]. Chronic accumulation of excess energy may result in increased adipose storage in the body. In animal and human investigations, there is consensus that the obesity phenotype is associated with increased SCFA in cecal and fecal samples compared to non-obese individuals. However, the population of the gut microbiota that may be associated with elevated cecal or fecal SCFA is widely debated.

Increased SCFA production in obese phenotypes will depend on a number of factors, including substrate availability, gastrointestinal transit, mucosal absorption, gut health, gut microbiota production, and symbiotic relationships between various groups of gut microbiota [11]. By producing SCFA, the gut microbiota significantly facilitates the metabolism of energy. SCFAs are microbial waste products produced by microbes in order to maintain gastrointestinal homeostasis. The production of SCFAs, stimulated by the consumption of dietary fiber and resistant starch, can enhance insulin sensitivity, promote glucose homeostasis, and have beneficial effects on body weight, lipid metabolism, and overall insulin sensitivity in humans [12]. SCFAs promote the production of the hormone peptide YY (PYY) by acting on the free fatty acid receptor 2 [12]. In addition to regulating satiety and suppressing appetite, SCFAs promote the production of PYY. PYY is secreted by neuroendocrine cells located in the ileum and colon in response to a meal, and this substance has been demonstrated to decrease appetite while inhibiting gastric motility and suppressing pancreatic secretion. According to previous research, butyrate supplementation reduces insulin resistance and protects against obesity caused by a high-calorie diet. The diversity of the gastrointestinal microbiota can be increased by increasing butyrate production via the consumption of superior whole cereals or bran [13].

The ratio of Firmicutes to Bacteroidetes (F:B ratio) predicts an individual's propensity for obesity or an increase in body fat [14]. Even when both groups (obese and non-obese) consume the same amount of food and energy, the obese microbiota has a significantly higher F:B ratio and a greater prevalence of Bacteroidetes. Compared with lean individuals, obese individuals have 90% more Bacteroidetes and 90% more Firmicutes [15], and the proportion of Firmicutes decreases as weight is lost.

Fasting, the type and caloric content of a meal, the use of antibiotics, age, location, the amount and frequency of physical activity, and genetic, technical, and clinical factors are among the complicated variables that may alter the composition of the intestinal microbiota. The gut microbiota has been connected to variations in dietary composition in uncontrolled overweight and obese adults. Variations in gastrointestinal microbiota have been identified, although only at the genus and family levels. Recent research on adolescent populations in Korea revealed that, despite having higher levels of *Prevotella/Prevotellaceae*, the normal-weight group had higher levels of *Bacteroides/Bacteroidaceae* [3]. The relative abundance of Firmicutes, Bacteroidetes, and Proteobacteria did not change significantly.

Inflammation

Recent research demonstrates a significant relationship between the metabolic and immune systems [16]. The release of inflammatory chemicals from adipose tissues, such as tumor necrosis factor-alpha (TNF-α), interleukin (IL)-6, and leptin, contribute to chronic inflammation in the context of obesity [17]. These substances can promote cancer development and increase the risk of colon, esophageal, and hepatocellular malignancies. Particularly elevated levels of leptin are associated with liver malignancy [17]. Additionally, adipose tissue secretes substances that increase the risk of cardiovascular disease [18]. These inflammatory chemicals activate processes that result in inflammation and an increase in adipose production. Obesity is characterized by persistent low-grade inflammation generated by these inflammatory chemicals. Maintaining a healthy balance between the body and bacteria is dependent on the intestinal mucosa [19]. Certain microorganisms in obese individuals continuously emit substances that stimulate a robust immune response, leading to inflammation. Increased gastrointestinal permeability and decreased production of specific fatty acids permit these substances to enter the bloodstream, resulting in chronic inflammation and metabolic problems [5].

By absorbing LPS, a component of the outer membrane of Gram-negative bacteria, the gastrointestinal microbiota contributes to chronic low-grade inflammation and obesity. This mechanism is essential for the development of persistent low-grade inflammation, a crucial aspect of obesity. Cani et al. demonstrated the connection between LPS and metabolic disorders by injecting bacterial LPS into germ-free mice and causing metabolic endotoxemia, which is characterized by elevated LPS levels in the circulation and resembles obesity-causing high-fat diets [19].

Additional research employing animal models, specifically toll-like receptor-4 (TLR4) mutant mice with impaired mucosal immunity, has elucidated the relationship between LPS and obesity [20]. These mice were fed high-fat diets, which led to an increase in body fat mass and LPS levels in the liver, adipose tissue, and muscle tissue. This syndrome, known as "metabolic endotoxemia," has been linked to a decrease in beneficial bacterial species, including *Bacteroides*, *Bifidobacterium*, and *Eubacterium* rectale-*Clostridium coccoides* group [21]. LPS acting on the CD14 receptor trigger inflammation and oxidative stress markers in visceral adipose tissue. CD14-deficient mice have been demonstrated in studies to reduce diet-induced obesity and inflammation [22]. By promoting the attachment of Gram-negative bacteria to the intestinal mucosa, high-fat diets increase LPS levels. Consequently, bacteria may infiltrate the bloodstream and collect in the lymph vessels of the mesentery. This allows bacteria to infiltrate the bloodstream and accumulate in the lymph nodes of the mesentery.

LPS can be absorbed by enterocytes and transported via chylomicrons and the lymphatic system to the liver and adipose tissue. LPS-induced metabolic endotoxemia promotes adiposity and insulin resistance. Studies on germ-free rodents demonstrated that LPS infusion causes metabolic endotoxemia symptoms, increased body fat, and weight gain comparable to that caused by high-fat diets [23]. Diets high in fat raise LPS levels, although to a lesser degree than septicemia or infection. Exposure to LPS decreases beneficial bacteria such as *Bacteroide*s and *Bifidobacterium*, which are essential for regulating LPS levels and enhancing mucosal barrier function. *C. coccoides*, a Gram-positive bacterium, is also inhibited by LPS [14]. These results demonstrate the complex relationship between intestinal microbiota, LPS absorption, and metabolic disturbances in obesity.

Antibiotic Use

It has been demonstrated that the gastrointestinal microbiota of young infants taking antibiotics changes over time, with an increase in *Bacteroides* and a decrease in *Bifidobacterium* [24]. Antibiotics that alter the gastrointestinal microbiota, such as norfloxacin and ampicillin, modify the expression of hepatic and intestinal genes implicated in inflammation and metabolism, thereby altering the hormonal, inflammatory, and metabolic milieu of the host [25]. These antibiotic-induced alterations may increase the prevalence of childhood obesity and overweight by fostering "obesogenic-bacterial growth." The development of infants' gut microbiota and their propensity for overweight and obesity in later childhood are linked, with significantly lower fecal *Bifidobacterium* and *Bacteroides* counts in overweight and obese pregnant women compared to those of normal weight pregnant women, and significantly higher *Escherichia coli* and *Staphylococcus aureus* counts [4]. Antibiotic-induced dysbiosis may result in weight gain and increased very low-density lipoprotein/high-density lipoprotein ratios [26].

Fat Storage

Researchers discovered that gut microbiota has the ability to influence fat accumulation [27]. This is mediated through various mechanisms. One of these mechanisms involves the gut microbiota increasing the absorption of glucose in the host's intestine and raising the serum glucose levels, which leads to higher expression levels of key transcription factors involved in fat synthesis in the liver, namely carbohydrate response element binding protein (ChREBP) and sterol regulatory element binding protein (SREBP)-1. These transcription factors promote fat synthesis [1].

Triglycerides synthesized in the liver are then transported to the bloodstream and taken up by fat cells with the help of lipoprotein lipase (LPL). Another important player in fat metabolism is the peroxisome receptor-activated proteins (PPARs), which control the expression of fasting-induced adipocyte factor, also known as fasting-induced adipocyte factor/angiopoietin-like protein 4 (FIAF/ANGPTL4). FIAF inhibits the activity of lipoprotein lipase (LPL), thereby preventing excessive fat accumulation in peripheral organs [28]. FIAF is produced by large intestine epithelial cells and the liver.

Interestingly, the gut microbiota can inhibit the expression of FIAF, leading to increased LPL activity and potentially promoting obesity in the host. Studies have shown that germ-free mice lacking the FIAF gene tend to have higher fat deposition compared to their littermates that express the gene [29]. The regulation of fat storage by FIAF/ANGPTL4 is influenced by the gut microbiota, and altering the composition of the microbiota can impact its expression and function.

**Figure 1:**
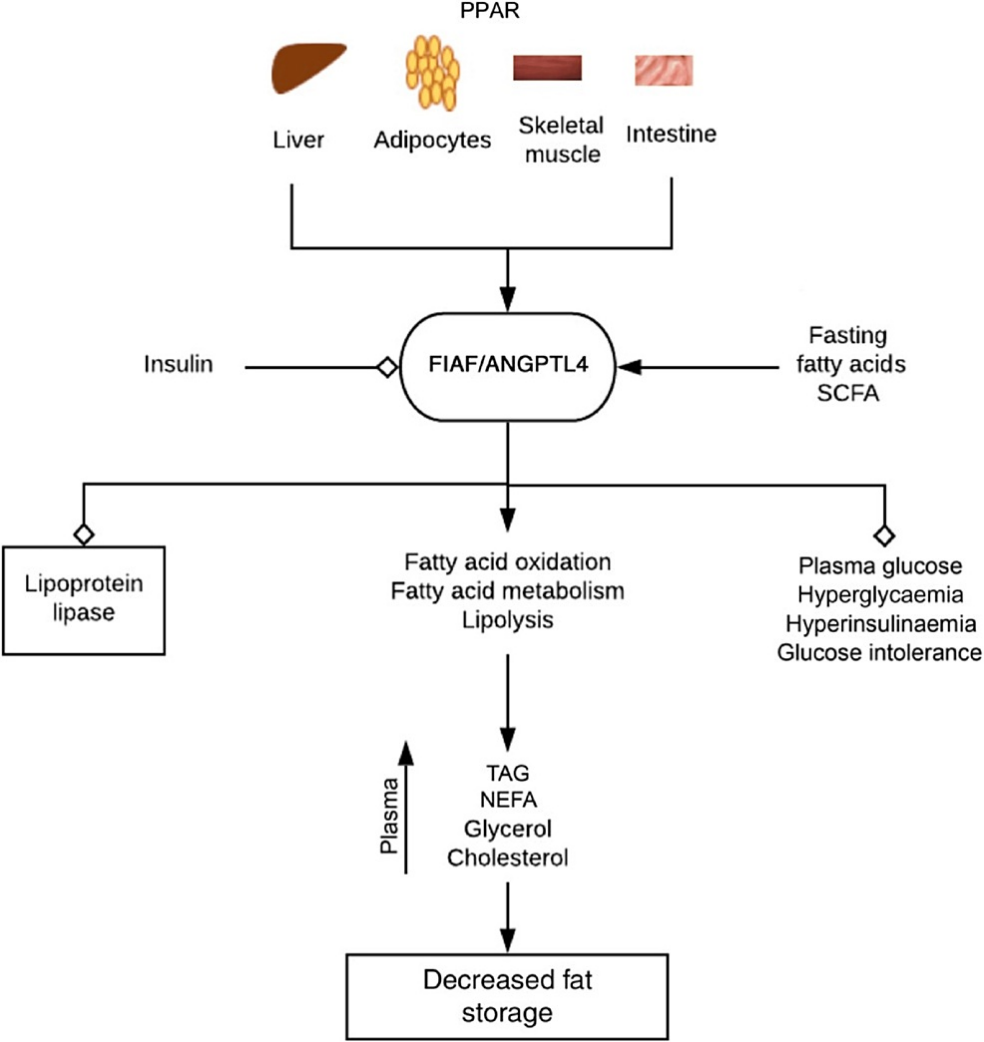
FIAF is triggered by fasting, fatty acids, and bacterial fermentation products, such as SCFA. It hinders lipoprotein lipase and stimulates the oxidation, metabolism, and breakdown of fatty acids. Consequently, it reduces fat storage, body weight, and obesity, while also lowering plasma glucose levels and enhancing insulin sensitivity. Insulin regulates FIAF, and it collaborates with ANGPTL4 in this process. FIAF: fasting-induced adipocyte factor; SCFA: short-chain fatty acids; ANGPTL4: angiopoietin-like protein 4; PPAR: peroxisome proliferator-activated receptors; TAG: triacylglycerol; NEFA: non-esterified fatty acids

For instance, supplementing mice with a high-fat diet with *Lactobacillus paracasei* F19 increased plasma levels of FIAF/ANGPTL4. Similarly, *Bacteroides thetaiotaomicron* showed no impact whereas *Bifidobacterium lactis* BB12 and *L. paracasei* F19 increased ANGPTL4 in colon cancer cells. The gut microbiota's ability to influence FIAF/ANGPTL4 expression raises the possibility of weight management therapies that alter ANGPTL4 via microbiota modification [30]. Figure 1 shows the role of FIAF in metabolic regulation and obesity.

Environmental Factors

Even though genes are essential for intestinal microbiota, environmental factors have a greater impact. In a study of 1,000 Israelis with relatively similar dietary and lifestyle preferences from around the world, it was discovered that the microbiome was unrelated to the participants' ancestry [31]. The heritability of the microbiome as a whole may be less than 2%. Food, medications, and anthropometric measurements account for more than 20% of the variance in the microbiome.

Diet is one of the primary factors contributing to obesity. The eating habits of industrialized nations and regions have become increasingly characterized by the consumption of large quantities of sugar and fat, leading to a steady rise in obesity. Because gut microbes rely on their hosts' food for survival and energy, dietary alterations have a significant effect on the gut microbiota. For example, when mice were fed high-fat diets, populations of Firmicutes and Pseudomonadota increased while populations of Bacteroidetes decreased. These changes were observed in mice resistant to weight gain, providing further evidence that dietary fat directly impacts the microbiome [32]. Insufficient sleep is another contributor to obesity. Insomnia disrupts circadian rhythms, which may alter the intestinal flora and increase the risk of obesity. Chronic sleep deprivation resulted in increased food intake and reversible alterations to the intestinal microbiota, including an increased abundance of *Ruminococcus* and *Prevotella *and a reduced number of Lactobacillaceae. These factors influence insulin sensitivity and lead to inflammation of visceral and systemic white adipose tissue. Stress makes people hungrier by activating genes that regulate metabolism, and it also increases their propensity to consume sugary and fattening foods, leading to obesity. Stress has a significant influence on the microbiota-gut-brain axis at all stages of life. Regardless of diet, the diversity of the gut microbiota increased, 50% of identified genera changed, the abundance of *Bacteroides* decreased, and the number of less dominant taxa increased in response to stress [33]. Obesity may be caused by substance abuse, mental health issues, and poor lifestyle choices, such as inactivity. Alternatively, because the gut microbiota is a "migrant" and is acquired from the environment, environmental influences have a bigger impact on it and may alter the prevalence and development of obesity.

Effect of probiotics, prebiotics, and other interventions

In the past decade, probiotics and prebiotics gained popularity for their potential benefits in preventing and treating chronic diseases [34]. Synbiotic supplements, which combine probiotics and prebiotics, are frequently used as health-promoting functional foods. Emerging research suggests that probiotics, which are living microorganisms, can improve the microbiome's functionality [35].

Probiotics

Probiotics are live bacteria, such as *Lactobacillus* and *Bifidobacterium*, that can benefit the host's health by restoring the equilibrium of bacteria in the colon. It has been discovered that they increase the number of beneficial bacteria that produce SCFAs while decreasing the number of detrimental bacteria that produce LPS [36]. Probiotics can reduce tissue damage, inflammation, and the presence of certain metabolites associated with opportunistic pathogens in this manner.

Additionally, probiotics have been demonstrated to have additional health benefits. They are capable of inhibiting fat accumulation, reducing inflammation and insulin resistance, and regulating various peptides in the gastrointestinal system [37]. Several studies have shown that probiotics can reduce BMI and total body fat, especially visceral fat [38]. In a study conducted on healthy males consuming a high-fat diet, probiotics were found to prevent weight gain and increased body fat.

A meta-analysis focusing on overweight or obese individuals confirmed probiotics' beneficial effects. Compared to the control group, individuals who received probiotics experienced significant reductions in body weight, BMI, waist circumference, and fat mass percentage [39]. Notably, the analysis revealed that higher probiotic concentrations, probiotics containing a single strain, and probiotics in food form were associated with larger fat mass reductions. In addition, the probiotic groups demonstrated lower insulin levels than the control group. The evidence suggests that probiotics can aid in weight loss while improving a variety of metabolic parameters.

According to Okeke et al. [36], probiotics containing *Lactobacillus* and *Bifidobacterium* may enhance Gram-negative gut bacteria by preventing the proliferation of harmful bacteria. Probiotics may also reduce gastrointestinal permeability and strengthen the intestinal epithelial barrier, which is essential for preventing inflammation and endotoxemia caused by the introduction of foreign objects into the blood [40]. SCFA is indispensable to this process. In addition, food treatment has a substantial beneficial effect on the host because it can alter the composition and function of intestinal microorganisms [41].

In a study by Qian et al. [42], a low-fat diet was substituted for a high-fat diet, and participants were randomly assigned to one of four groups: High-fat diet alone, dietary intervention alone, high-fat diet with probiotics (*L. acidophilus*, *Bifidobacterium longum*, and *Enterococcus faecalis*), or dietary intervention with probiotics. Four months later, fecal samples were collected and 16S rDNA (ribosomal DNA) sequencing was conducted to investigate the specific changes in the microbiota composition caused by the combination of probiotics and the dietary intervention. According to the results, the two butyrate-producing families Ruminococcaceae and Lachnospirae exhibited greater microbial diversity when probiotics and dietary intervention were administered together as opposed to separately. A diminished F:B ratio was also observed in the intestinal microbiome of individuals with obesity caused by a high-fat diet. However, it is vital to emphasize that this ratio alone is not a direct cause of human obesity, but it may be impacted by dietary choices. It appeared that the combination of probiotics and dietary intervention increased the presence of beneficial bacterial species and decreased the abundance of certain detrimental species within the microbiota. This suggests that the use of probiotics in conjunction with dietary intervention may provide a natural approach to combating obesity caused by a high-fat diet [33].

Prebiotics

Previously, it was believed that prebiotics were undigested food components that reached the intestinal lumen and were utilized selectively by beneficial microbes in the host. Recent research has focused heavily on inulin, galacto-oligosaccharides (GOS), and fructo-oligosaccharides (FOS) [43]. Inulin, a fermentable carbohydrate, was found to increase the density of cells producing the appetite-suppressing hormone PYY by 87%, indicating its potential function in reducing calorie intake and enhancing obesity treatment [40].

Koutnikova et al. [41] found that supplementation with GOS increases the levels of *Bifidobacterium* spp. and decreases the levels of *Bacteroides* in healthy individuals. It has also been discovered that prebiotics alter the composition and function of the intestinal microbiota. They increase the number of butyrate-producing bacteria like *Bifidobacterium* spp, which can improve metabolic outcomes and strengthen the intestinal barrier against infections [41]. In addition, research has shown that the presence of FOS increases *Bifidobacterium* and *Lactobacillus* spp, resulting in decreased levels of the appetite hormone ghrelin, increased levels of PYY, and decreased food intake [38].

Wiese et al. confirm that FOS can promote the proliferation of *Bifidobacterium* spp, also known as bifidogenesis. In a randomized, double-blind, placebo-controlled study, human participants were administered FOS at varying doses (2.5, 5, and 10 g/d) or a placebo (maltodextrin 10 g/d), and fecal samples were collected at specific time points to analyze microbial [43]. The consumption of FOS increased the number of *Lactobacillus* and *Bifidobacterium* spp.

In addition, higher doses of FOS promoted the expansion of *Lactobacillus*-related phyla (Firmicutes). However, the abundance of this phylum decreased following the cessation of prebiotic usage. Several butyrate-producing bacteria, such as *Faecalibacterium*, *Ruminococcus*, and *Oscillospira*, were also significantly altered [43]. These findings suggest that ingesting prebiotics promotes microbial diversity and is beneficial for the host.

Additionally, a single dose of prebiotic therapy decreases the leptin resistance caused by a high-fat diet. Studies on *Bifidobacterium* and GOS alone and in combination as synbiotics showed no additional benefits. Ongoing clinical studies are examining its potential use in the treatment of obesity. Several studies indicate that lycopene, dark chocolate, and cocoa flavanols can function as prebiotics and have an effect on intestinal flora. According to research conducted on obese participants, daily lycopene consumption for one month, with or without dark chococlate, induced substantial alterations in the intestinal flora [43]. The lycopene formulations induced a dose-dependent increase of predominantly *Bifidobacterium* spp. However, *Lactobacillus* levels increased in the dark chocolate formulation. In addition, these formulations reduced the quantity of Bacteroidetes significantly.

Synbiotics and Antibiotics

Synbiotics, which consist of probiotics and prebiotics, have demonstrated greater health benefits than probiotics or prebiotics alone. Studies indicate that oligofructose-enriched inulin, in conjunction with *Lactobacillus rhamnosus* and *Bifidobacterium lactis*, has the potential to modify the gut microbiota by promoting the growth of beneficial bacteria such as *Lactobacillus* and *Bifidobacterium* while inhibiting the growth of *Clostridium perfringens* [44]. However, additional research is required to validate these results in humans.

Broad-spectrum antibiotics have shown potential in reducing insulin resistance in obesity-related organs such as the liver, muscle, and adipose tissue in animal models. Additionally, it can reduce intestinal permeability and reduce inflammation caused by LPS [45]. Reducing circulating LPS levels and inhibiting TLR4 activation are methods that can be used to achieve these results. In addition, the administration of antibiotics increases portal acetate levels, which activate pathways that increase energy expenditure. However, transforming these discoveries into human applications is difficult due to concerns about antibiotic resistance, changes in commensal bacteria, and possible associations with weight gain [46].

Probiotics may modulate the gut microbiota and aid in weight loss by enhancing satiety, enhancing glucose tolerance, reducing inflammation, promoting leptin and insulin sensitivity in the hypothalamus, and enhancing gut permeability [47]. It has also been demonstrated that bariatric surgery decreases the F:B ratio by decreasing Firmicutes and increasing Bacteroidetes, resulting in an increase in microbial diversity in the intestine [48].

Diet

Different diets have different effects on the composition and diversity of the intestinal microbiome, which can impact the development of inflammatory diseases in adults [49]. To fathom the effects of diets on obesity, it is essential to comprehend the relationship between diet, intestinal microbiota, and body weight.

Microbial dysbiosis has resulted from the Westernization of dietary habits [50]. African children who ingest low-fat, high-fiber diets have greater microbial diversity and fewer hazardous bacteria than their European counterparts, who have higher levels of Firmicutes and Enterobacteriaceae (such as *Shigella* and *Escherichia*). In contrast, a high-fat, low-fiber diet decreases beneficial gastrointestinal flora, SCFAs, and microbial diversity [51]. Independent of calorie intake, the consumption of high-fiber foods such as fruits, vegetables, and legumes has been associated with reduced weight gain and increased microbial diversity.

In obese mice fed a high-fat diet, the medicinal fungus *Antrodia cinnamomea* and the antioxidant astaxanthin, derived from carotenoids, improved the intestinal microbiome. These interventions increase the F:B ratio and boost *Akkermansia* abundance [52]. Consequently, they effectively reduce weight gain, enhance lipid and glucose metabolism, and regulate the intestinal microbiome.

Exercise

The gut microbiota is influenced by physical activity, resulting in modifications to its composition and activity. Aerobic exercise has been shown to enhance the degradation of macromolecules and increase microbial diversity in the gastrointestinal microbiomes of athletes, with a focus on butyrate-producing bacteria [53]. According to research, increasing microbial diversity requires both sufficient fiber consumption and moderate-to-vigorous physical activity. In addition, aerobic exercise has been associated with an increase in Bacteroidetes and a decrease in Firmicutes in obese individuals. Alterations in the intestinal microbiota induced by exercise are independent of dietary factors and instead depend on obesity status and exercise adherence [54].

## Conclusions

The prevalence of obesity among both adults and adolescents has reached epidemic proportions. Obesity is influenced by a variety of factors, including genetics, environment, lifestyle, and diet, but the precise causes remain unclear. The gastrointestinal microbiota may be a factor affecting energy balance and metabolism. According to studies, eating habits may have a significant effect on the composition of gut flora, promoting an obese phenotype. A high-fat, high-carbohydrate diet induces a decrease in beneficial bacteria and an increase in pathogenic bacteria. Inadequate gut barrier function, inflammation, dyslipidemia, and hormonal imbalances induced by these alterations in the composition of the microbes eventually lead to obesity, diabetes, and cancer. It is intriguing that probiotics, prebiotics, and symbiotic therapies have the potential to regulate the intestinal microbiota and reduce the risk of obesity. Although studies on animals have yielded insightful information, additional research is necessary to confirm these results in humans.
